# Minimal Residual Disease Detection and Evolved *IGH* Clones Analysis in Acute B Lymphoblastic Leukemia Using *IGH* Deep Sequencing

**DOI:** 10.3389/fimmu.2016.00403

**Published:** 2016-10-04

**Authors:** Jinghua Wu, Shan Jia, Changxi Wang, Wei Zhang, Sixi Liu, Xiaojing Zeng, Huirong Mai, Xiuli Yuan, Yuanping Du, Xiaodong Wang, Xueyu Hong, Xuemei Li, Feiqiu Wen, Xun Xu, Jianhua Pan, Changgang Li, Xiao Liu

**Affiliations:** ^1^BGI-Shenzhen, Shenzhen, China; ^2^China National Genebank-Shenzhen, BGI-Shenzhen, Shenzhen, China; ^3^Hematology and Oncology Department, Shenzhen Children’s Hospital, Shenzhen, China; ^4^KingMed Diagnostics, Guangzhou, China; ^5^Department of Biology, University of Copenhagen, Copenhagen, Denmark

**Keywords:** acute B lymphoblast leukemia, minimal residual disease, high throughput sequencing, *IGH*, clonal evolution

## Abstract

Acute B lymphoblastic leukemia (B-ALL) is one of the most common types of childhood cancer worldwide and chemotherapy is the main treatment approach. Despite good response rates to chemotherapy regiments, many patients eventually relapse and minimal residual disease (MRD) is the leading risk factor for relapse. The evolution of leukemic clones during disease development and treatment may have clinical significance. In this study, we performed immunoglobulin heavy chain (*IGH*) repertoire high throughput sequencing (HTS) on the diagnostic and post-treatment samples of 51 pediatric B-ALL patients. We identified leukemic *IGH* clones in 92.2% of the diagnostic samples and nearly half of the patients were polyclonal. About one-third of the leukemic clones have correct open reading frame in the complementarity determining region 3 (CDR3) of *IGH*, which demonstrates that the leukemic B cells were in the early developmental stage. We also demonstrated the higher sensitivity of HTS in MRD detection and investigated the clinical value of using peripheral blood in MRD detection and monitoring the clonal *IGH* evolution. In addition, we found leukemic clones were extensively undergoing continuous clonal *IGH* evolution by variable gene replacement. Dynamic frequency change and newly emerged evolved *IGH* clones were identified upon the pressure of chemotherapy. In summary, we confirmed the high sensitivity and universal applicability of HTS in MRD detection. We also reported the ubiquitous evolved *IGH* clones in B-ALL samples and their response to chemotherapy during treatment.

## Introduction

Acute lymphoblastic leukemia (ALL) is one of the most common malignant disease in children worldwide and represents approximately a quarter of cancer diagnoses among children younger than 15 years. During the past 25 years, there has been a gradual increase in ALL incidence according to the National Cancer Institute’s Surveillance, Epidemiology, and End Results (SEER) Program, as well as published reports (1). About 85% of pediatric ALL is classified as B cell lineage ALL (B-ALL). With the improvement of treatment strategies, the 5-year survival rate of ALL has greatly increased from 57.2% during 1975–1977 to 91.8% during 2004–2010 according to the cancer statistics by National Institutes of Health (NIH). In spite of increased cure rates in pediatric ALL, relapse still occurs in approximately 15–20% of patients (2), and minimal residual disease (MRD) is the main risk factor for ALL relapse. Therefore, MRD level has become an important clinical index for doctors to assess treatment response, to adjust the treatment strategy during ALL therapy, and to predict relapse after treatment (3–8). On the other side, some patients are likely to be over-treated and experience serious side effects of chemotherapy. Thus, it is important to consider increasing cure rates together with decreasing toxicity during treatment, and monitoring MRD can provide great help.

Flow cytometry (FCM), fusion genes analysis, and molecular analysis of immunoglobulin (Ig) or T cell receptor (TCR) gene rearrangements are three principal methods for MRD detection in childhood ALL. FCM is a method based on the immunophenotype, which distinguishes the ALL cells from the normal leukocytes by the cell markers they expressed (9). FCM has become a widely used method for clinical investigations due to the rapidity of method, but the immunophenotypic shifts induced by chemotherapy could affect the confidence and accuracy of MRD detection (10, 11). Detection of MRD based on the gene fusions caused by chromosomal translocation can achieve very high sensitivity, but this method only applicable to ALL patients with chromosomal translocations that are identified only in a minority of patients (12). Another ALL MRD detection method is based on the antigen receptor gene rearrangement by real-time quantitative polymerase chain reaction (RQ-PCR) (13). This method is also very sensitive (14), but is laborious and time-consuming due to the need to design patients-specific primers (15). Besides, false-negative result due to clonal evolution is a major problem using unique Ig or TCR rearrangement as RQ-PCR target for MRD detection (7). Several studies have reported the application of high throughput sequencing (HTS) of TCR repertoire or Ig repertoire in MRD detection (16–18). Bone marrow (BM) is the most used specimen for MRD detection in leukemia; however, it has been proposed that peripheral blood (PB) might represent a more convenient specimen for monitoring MRD. For acute myeloid leukemia (AML), Maurillo et al. showed that PB could be used as the cell source for MRD detection using FCM, and MRD levels in PB were positively correlated with MRD measured in BM (19). Coustan-Smith et al. (20) demonstrated PB may be used to monitor MRD in T-lineage ALL patients. By contrast, in B-lineage ALL patients, PB did not work well for MRD detection, but still provided valuable prognostic information (20). However, these studies did not analyze and describe the feasibility and clinical significance of MRD detection using PB by HTS.

Tumors, including hematological tumors, are composed of heterogeneous subpopulations, and tumors with higher heterogeneity had higher rates of resistance to chemotherapy compared with tumors with lower phenotypic variability (21). Some of the subpopulations were produced due to on-going cancer clonal evolution in disease development and disease treatment. The massive evolution of the leukemic cell plays a pivotal role in disease progression and relapse (22), and a prior study reported the mechanisms of clonal evolution in B-ALL (23). The evolution related to V gene replacement in pre-treatment B-ALL samples and the clinical significance has been reported by several studies (17, 24). However, to our knowledge, the extent and dynamics of the clonal evolution of leukemic cells during treatment, as well as its clinical significance, have never been reported.

In this study, we investigated the immunoglobulin heavy chain (*IGH*) repertoires of 51 B-ALL patients before and during the chemotherapy using HTS technology. We identified disease-bearing *IGH* rearrangements in 92.2% of the patients and half of the patients harbored two or more leukemic *IGH* clones. Those clonal B cells were in the early developmental stages. We also analyzed the *IGH* repertoires of nine diagnostic samples in the RNA level and the result showed that all the disease-causing clones, including those without function, can transcribe mRNA. Next, we demonstrated the high sensitivity of HTS technology in MRD detection and the prognostic value of MRD detection using PB samples. Lastly, we analyzed the evolved *IGH* clones induced by V gene replacement, and at the same time evaluated the changes of the numbers and the frequencies of those evolved *IGH* clones during treatment.

## Materials and Methods

### Patients and Samples

We studied 51 childhood B-ALL patients diagnosed in Shenzhen Children’s Hospital. BM specimens and (or) PB were obtained at diagnosis and during treatment, and in total 169 specimen were collected (Table S1 in Supplementary Material). The study was carried out in accordance with the recommendations of Declaration of Helsinki and was approved by BGI-IRB. Written informed consent was obtained from the parent(s) or guardian(s) of each child. All the BM and PB specimens were collected in heparin and stored at −80°C until analysis was conducted. High-quality gDNA were extracted from the frozen BM and PB samples using DNA Blood mini kit (QIAGEN, Cat. no.51106).

### Disease Risk Stratification

B-ALL patients were stratified into three risk groups according to the following criteria:

Standard risk (SR): (1) age at diagnosis between 1 and 6 years; (2)WBC < 20 × 10^9^/L; (3) good prednisone respond (GPR) at 7 days treatment, peripheral blasts < 1.0 × 10^9^/L at day 8; (4) BM aspiration results M1 (blasts < 5%) or M2 (blasts 5% ~25%) at day 15 post induction; and (5) BM aspiration results M1 at day 33 post induction.

Intermediate risk (IR): (1) age at diagnosis <1 year or ≥6 years; (2) WBC ≥ 20 × 10^9^/L; (3) GPR; (4) BM aspiration results M1 or M2 at day 15 post induction; (5) BM aspiration results M1 at day 33 post induction; (6) reach to SR but BM aspiration results M3 (blasts > 25%) on day 15 of induction therapy, and BM aspiration results M1 on day 33 of induction therapy.

High risk (HR): (1) IR but BM aspiration results M3 on day 15 of induction therapy; (2) poor prednisone respond (PPR), peripheral blasts > 1.0 × 10^9^/L at day 8; (3) BM aspiration results M2 or M3 at day 33 of induction; (4) *t*(9:22) (BCR/ABL) positive or *t*(4:11) (MLL/AF4); (5) testicle leukemia at diagnosis and did not clinically resolve by day 33 of chemotherapy; (6) large mediastinum mass at diagnosis, and did not completely resolve by day 33; (7) central nervous system leukemia (CNSL) at diagnosis.

### Immunophenotyping and MRD Detection by FCM

Immunophenotyping and MRD detection by FCM was performed at KINGMED CENTER FOR CLINICAL LABORATORY. The red blood cells of BM samples were lysed to get nucleated cells. For MRD detection, BM nucleated cells were stained with the following monoclonal antibody combinations: (1) CD58 FITC, CD34 PE, 7AAD PerCP-CY5.5, CD10 PE-Cy7, CD19 APC, CD38 V450, CD45 V500; and (2) CD66c FITC, CD13+CD33 PE, CD34 PerCP-CY5.5, CD10 PE-Cy7, CD19 APC, CD15 V450, CD45 V500. The labeled cells were analyzed using a BD CANTO II flow cytometer (Becton Dickinson, San Jose, CA, USA) with at least 100,000 events were acquired. Data were analyzed using BD FACSDiva (Becton Dickinson) and FCS Express (*De Novo* Software, Los Angeles, CA, USA) software. The compensation matrix was set up using BD CompBeads (Becton Dickinson) for fluorochrome-conjugated antibodies. Quality control was performed using BD Cytometer Setup and Tracking Beads (Becton Dickinson).

### High Throughput Sequencing of *IGH* Repertoire

The complementarity determining region 3 (CDR3) of the variable regions of *IGH* was amplified by multiplex PCR. Concretely, the complete VDJ rearrangements of *IGH* were amplified from 1200 ng gDNA with 12 degenerate forward primers annealed to the 55 functional variable genes (V) and 4 reverse primers annealed to the 6 functional joining genes (J) listed in the IMGT database. The primers have been carefully evaluated to minimize PCR bias, and were listed in Table S4 in Supplementary Material. PCRs (50 μL) were set up at 25 μL of 2× QIAGEN Multiplex PCR master mix, 5 μL of QIAGEN Q solution, 1 μL of 10 μM forward primer pool, 1 μL of 10 μM reverse primer pool, and 18 μL of 67 ng/μL gDNA. The reaction cycling conditions were: 95°C for 15 min, 30 cycles at 94°C for 30 s, 60°C for 90 s, and 72°C for 30 s, followed by a final extension at 72°C for 5 min. The target amplified products (120–200 bp) was purified by electrophoresis on 2% agarose gel, and the Illumina Hiseq sequence adaptors were ligated. Then the sequencing libraries were sequenced with standard 2 × 150 paired end reads on Illumina Hiseq2500 platform.

### Analyses of the Sequencing Data

Sequencing data were analyzed by the TCR and BCR repertoire analyzing pipeline IMonitor (25). About 2–5 million mapped reads (include correct V and J genes) remained after the above pipeline for each sample, and we used a random subset of 2 million mapped reads for continued analysis. In order to remove the false *IGH* clones derived from sequencing error or contamination from other samples in the same Illumina sequencing lane, we filtered the clonotypes with supported reads lower than the inferred average sequencing depth for every B cells in each sample. The average sequencing depth was inferred as following: first, the total input cell number for amplification was 2 × 10^5^ cells, equating to 1200 ng input gDNA. Second, we calculated the B cell number by multiplying the total input cell number by the B cell percentage (detected by FCM according the CD19 marker). Third, the average sequencing depth was inferred by dividing the total used reads (2 million) by the B cell number used in the sample. The CDR3s were classified as non-functional if frame-shift mutations or stop codons existing in CDR3 region.

### MRD Detection by HTS

Similar to previous studies, we defined the CDR3s in diagnostic samples with frequency higher than 10% as leukemic clones (17, 18). In the post-treatment samples, we determined the MRD by calculating the frequencies of identical CDR3 sequences with the leukemic clones in the level of total nucleated cells. The MRD is calculated by multiplying the frequencies of leukemic clones by B cell percentage in the total nucleated cells, which is determined by FCM.

### Identifying the Evolved *IGH* Clones of Leukemic Clones

The method used by Gawad et al. (24) was used to identify the evolved *IGH* clones from the leukemic clones with minor modification. Concretely, the evolved *IGH* clones were defined as: (1) identical J sequence with the leukemic clone; (2) share at least eight bases of identical NDN sequence with the leukemic clone; (3) different V gene from the leukemic clone; (4) more than three mismatches in CDR3 sequence if the evolved *IGH* clone had the same length as the leukemic clone.

## Results

### Clinical Characteristics of the B-ALL Patients

We randomly, without any prior knowledge of the clonal *IGH* rearrangement existence, collected 51 pediatric B-ALL patients in total. According to the disease risk stratification criteria, 19 of them were classified as SR, 18 as IR, and 14 as HR (Table S1 in Supplementary Material, details in the section “Materials and Methods”). The characteristics and clinical information of the patients, including diagnostic age, gender, and cytogenetics were collected and listed in Table S1 in Supplementary Material. Ninety-six percent of patients (49/51) were aged between 1 and 10, and 2 patients were more than 10 years old. Among the patients, 35.3% (18/51) were females, and 23.5% (12/51) were diagnosed with TEL-AML1 gene fusion. All the 51 patients showed good treatment result after 2–3 months of chemotherapy.

### Clonal Leukemic *IGH* Identification in Pre-Treatment BM Samples

**Figure 1:**
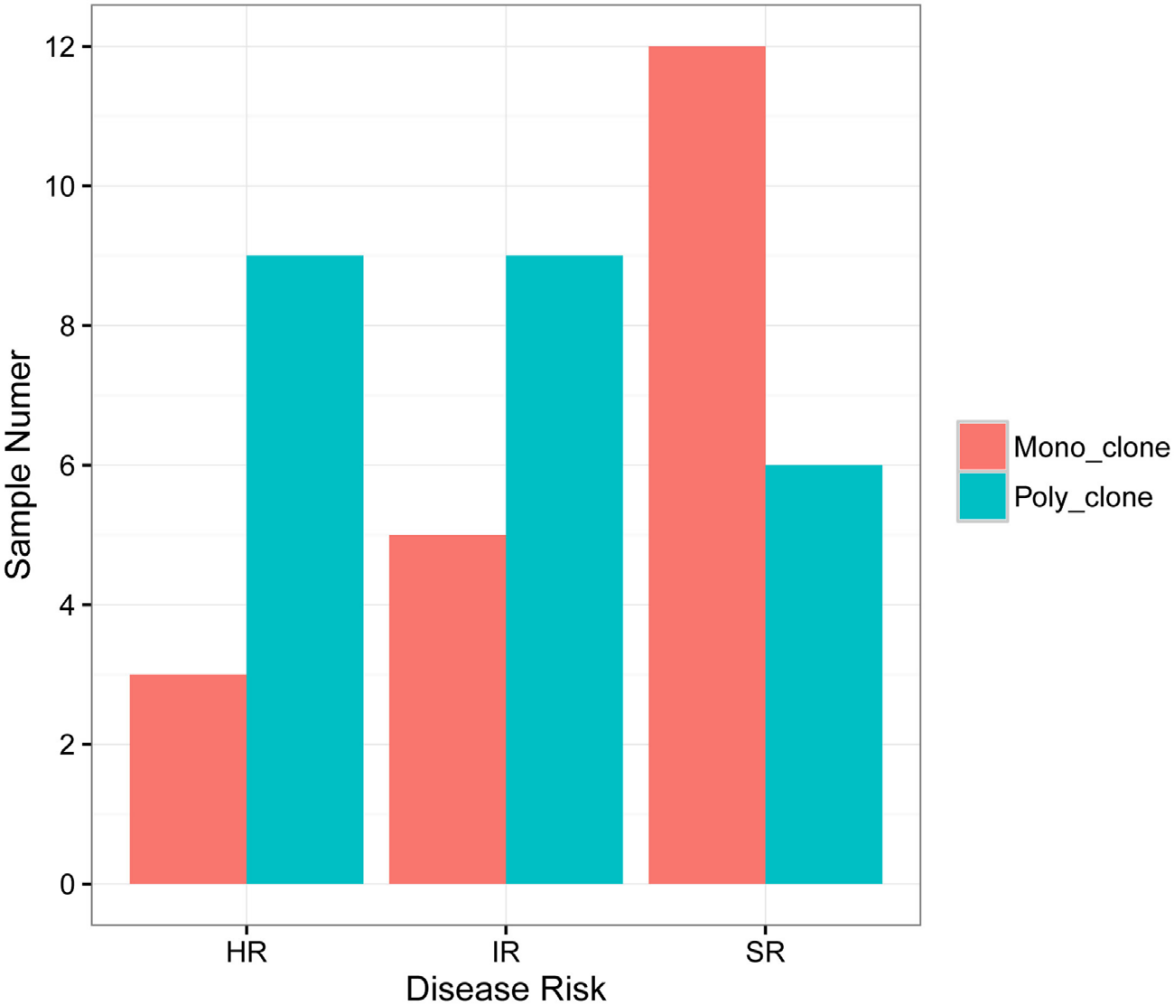
**Correlation of B-ALL disease risk with the number of leukemic clones in diagnostic sample**. HR, high risk; IR, intermediate risk; SR, standard risk; “mono_clone,” just one leukemic clone in the diagnostic sample; “poly_clone,” two or more leukemic clones in the diagnostic sample.

According to the definition of leukemic clones (see Materials and Methods), we identified leukemic *IGH* CDR3s with complete V–D–J rearrangement in a frequency of above 10% in the diagnostic BM samples in 47 of 51 patients (92.2%). Of the 47 patients with leukemic *IGH* clones, 24 had two or more leukemic clones (Table 1; Table S2 in Supplementary Material). Therefore, we identified 77 leukemic clones from the 47 patients in total (Table S2 in Supplementary Material). The four patients without identification of leukemic clones did not show any significantly distinct immunophenotypes. They may harbor incomplete VDJ rearrangement, or use the pseudo genes to recombine VDJ which cannot be amplified by our multiplex PCR primer set. We then investigated if the number of leukemic clones related with the disease risk. The result showed that more patients in SR group have just one disease clone; however, more patients in HR group have two or more disease clones (Figure 1).

**Table 1:** **Leukemic clones identification in 52 B-ALL patients**.

Disease *IGH* clone	Number of patients	Percentage
0	4	7.8
1	23	45.1
>1	24	47.1
Total	51	100

Because B-ALL is a kind of cancer originating from the immature stage of B cells, we investigated if the leukemic *IGH* rearrangements underwent positive selection. Of the 77 leukemic clones, 28 (about one-third) were functional with correct open reading frame (ORF) in the CDR3, and the other 49 were classified as non-functional due to the occurrence of frame-shift or stop-codon mutations in the CDR3 (Table S2 in Supplementary Material). The ratio of functional to non-functional clones was proportional with that expected by chance. The data above demonstrated that these B cells were immature precursor B cells without experiencing positive selection. We next compared the mRNA transcription of the functional and non-functional leukemic clones by sequencing the *IGH* repertoire of RNA extracted from the same BM specimen of nine patients. All the leukemic clones, including the non-functional clones, were detected in RNA with high frequency. Surprisingly, the transcription of non-functional leukemic clones was not eliminated or even decreased compared with the functional clones (Figure S1 in Supplementary Material), implying the resistance to the nonsense mRNA decay pathway (26, 27). However, compared with the frequencies in DNA samples, the frequencies of most leukemic clones in RNA decreased largely, both for functional and non-functional ones (Table 2), which could be due to decreased transcription of the pre-B cells (28), including B-ALL leukemic cells, and it is consistent with other report (29).

**Table 2:** **The frequency of leukemic clones in DNA and RNA of the same bone marrow samples**.

Patients	Leukemic clone ID	Leukemic clones frequency in DNA (%)	Leukemic clones frequency in RNA (%)
P001	P001B	35.79	10.94
	P001A	15.42	4.30
	P001D	14.82	3.07
	P001C	14.80	4.59
P005	P005	84.56	34.53
P006	P006	78.62	30.36
P007	P007A	45.52	27.39
	P007B	40.96	1.16
P008	P008	90.87	20.19
P013	P013	25.12	11.79
P011	P011A	62.80	32.14
	P011B	18.44	0.82
	P011C	11.60	17.85
P015	P015A	27.64	10.02
	P015B	10.59	4.30
P009	P009	87.59	19.61

*Underline in the clone frequency means those clones with correct ORF*.

### Comparison of MRD Results Detected by HTS with FCM in Follow-up BM Samples

With the precise *IGH* CDR3 sequence information of the leukemic clones, we determined the frequencies of the leukemic clones in the follow-up BM samples. MRD based on the total nucleated cells was calculated by multiplying the frequency of the leukemic clone by the percentage of the B cells in total nucleated cells, which was determined by FCM for those samples. MRD levels and leukemic clone frequencies decreased as treatment progressed (Table S2 in Supplementary Material and Figure S2 in Supplementary Material). With the purpose of evaluating the reproducibility of MRD detection by HTS, we performed two replicated MRD detections from equal BM specimen for three samples of two patients (P049-15, P049-33, and P051-15) and the result showed good reproducibility (Table S3 in Supplementary Material).

**Figure 2:**
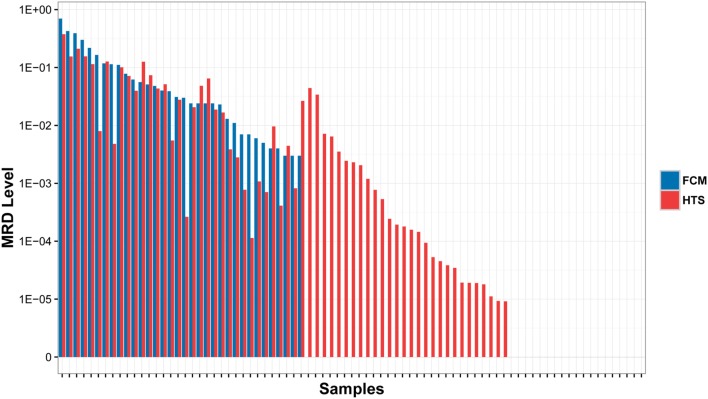
**Comparison of MRD results detected by high throughput sequencing (HTS) with flow cytometry (FCM) assays**. The samples were classified into three subsets: (1) MRD was detected positive by both HTS and FCM (left); (2) MRD were detected positive by HTS, but negative by FCM (middle); and (3) MRD was detected negative by both methods (right).

In order to assess the accuracy of the MRD detection by *IGH* repertoire HTS, we compared the detection results of HTS with the results detected by FCM, which were performed as the golden standard for MRD detection. All 34 MRD positive samples by FCM were detected as positive by HTS with similar MRD levels. Twenty-eight samples were detected as MRD positive by HTS, but MRD negative by FCM. The other 19 samples were MRD negative by both HTS and FCM. No MRD positive samples by FCM were detected negative by HTS, which demonstrated the higher sensitivity of HTS (Figure 2).

### Identification of Leukemic Clones and MRD Detection in the PB Samples

**Figure 3:**
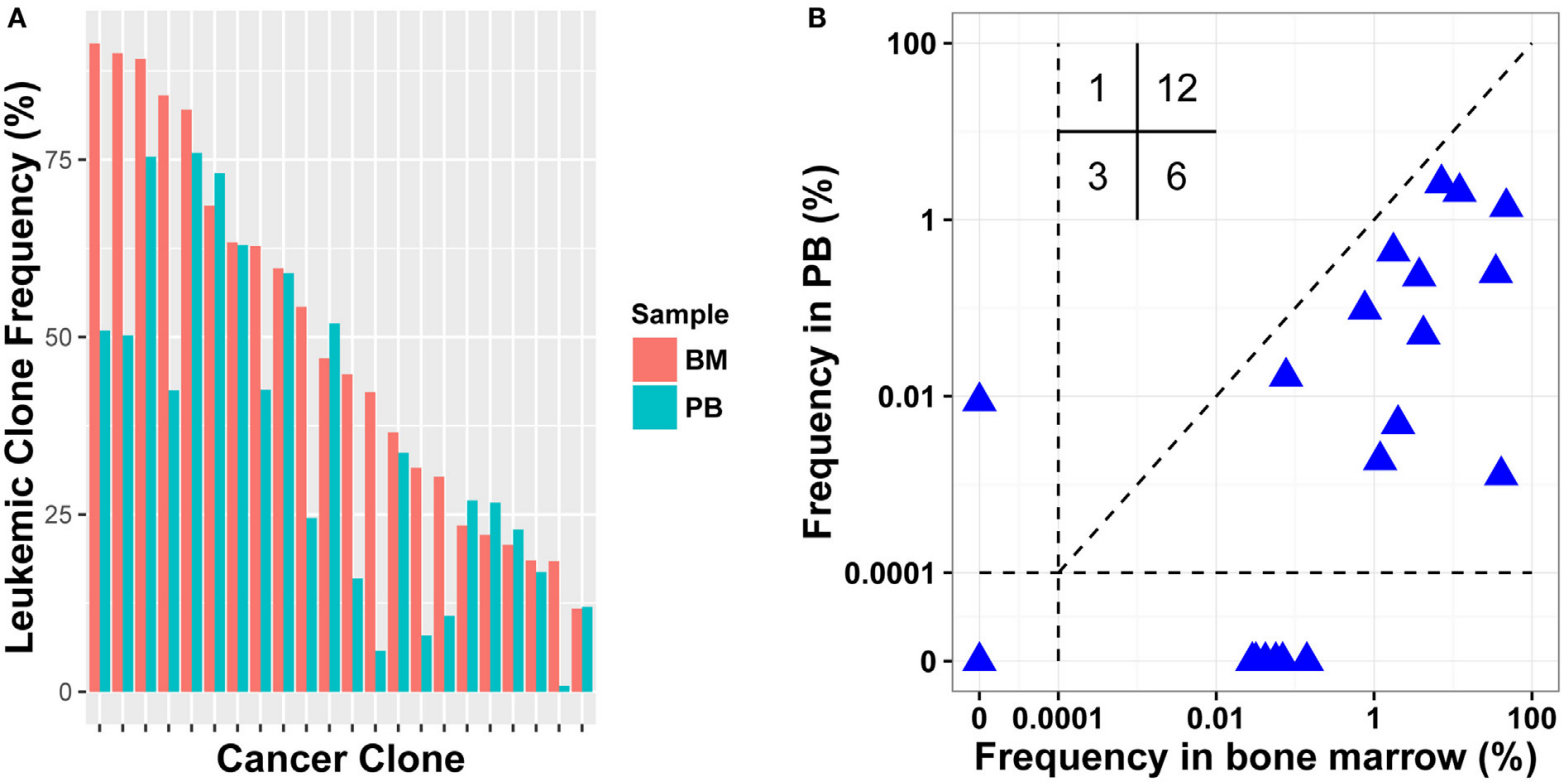
**Comparison leukemic clone frequency between bone marrow (BM) and peripheral blood (PB) specimens for diagnostic (A) and follow-up (B) samples**. Values in the up left in **(B)** represent the number of samples in each quadrant.

Because BM aspiration can be painful, using PB instead of BM was evaluated for MRD detection. For 15 patients, we collected both diagnostic BM and PB samples (Table S1 in Supplementary Material). We investigated if the leukemic clones identified in BM also existed in PB with high frequencies. The results revealed that all leukemic clones could be detected in PB with frequency of above 5%, except one clone, which was 18.405% in BM, but was just 0.810% in PB (Figure 3A). The Pearson correlation coefficient between the frequencies of leukemic clones in diagnostic PB and BM was 0.7860. Therefore, PB can be used instead of BM in disease diagnosis and in leukemic clone identification for most patients.

To test MRD detection in PB specimens and to compare it with BM, post-treatment BM and PB samples were collected simultaneously in 15 pairs from 11 patients (Table S1 in Supplementary Material). We determined the frequencies of leukemic clones using the same method. In total, 22 leukemic clones were identified in pre-treatment BM samples for the 11 patients, and 18 clones were positive in post-treatment BM. When detecting the 18 clones in the corresponding PB samples, 12 clones were detected and other 6 were PB negative. When evaluating their frequencies in BM, 11/12 PB positive clones had relatively higher frequency than the 6 PB negative clones. Three leukemic clones were MRD negative in both PB and BM; one was positive in PB with clonal frequency of less than 0.01%, but was negative in BM. We then assessed if the leukemic clone frequency in PB correlated positively with that in BM, and a weak correlation was found with a Pearson correlation coefficient of 0.322 (Figure 3B). Overall, although PB could be used to detect MRD to some extent, the sensitivity of MRD detection using PB was still lower than using BM and it resulted in false negative for some samples.

### Monitoring the Evolved *IGH* Clones of the Leukemic Clones Before and during Therapy

On-going change at the *IGH* gene is one of the most important ways leading to the diversity of cancer cells in B-ALL. After finishing *IGH* variable (V) gene, diversity (D) gene, and joining (J) gene rearrangement during B cell development (30), the V segments upstream of the used V gene are still reserved, which make the V gene replacement possible by rearranging an upstream V gene with the complete V–D–J rearrangement. Because the loss of 5′ recombination signal sequences (RSS) in D segment of the rearranged V–D–J exon, cryptic RSS within the used V gene is involved in V gene replacement, which effectively increases the length of the V–D–J junction of the new rearrangement (31). The identified evolved *IGH* rearrangements of leukemic clones in this paper could be generated by three different mechanisms. The first and second mechanisms are based on V replacement in *IGH* gene. The first mechanism is that the leukemic clone is the ancestral clonotype and the evolved *IGH* clones derived from continuous V replacement. In this situation, the V segment of the evolved *IGH* clones will be located at the upstream of the leukemic clone, and the CDR3 length of evolved *IGH* clones would be longer than that of leukemic clone (31). The second mechanism is that the leukemic clone is produced by V replacement from a more ancestral pre-leukemic clonotype. In this situation, the V segment of leukemic clone will located at the downstream of the ancestral clone, and the CDR3 length of the leukemic clone would be longer than the ancestral clone. The third mechanism is that the ancestral (pre-leukemic) clone finished an incomplete D–J rearrangement of *IGH* locus, and the leukemic clone and other evolved *IGH* clones underwent independent *IGH* V rearrangement. Therefore, the CDR3 length of the leukemic clone and the evolved IGH clones would be comparable.

**Figure 4:**
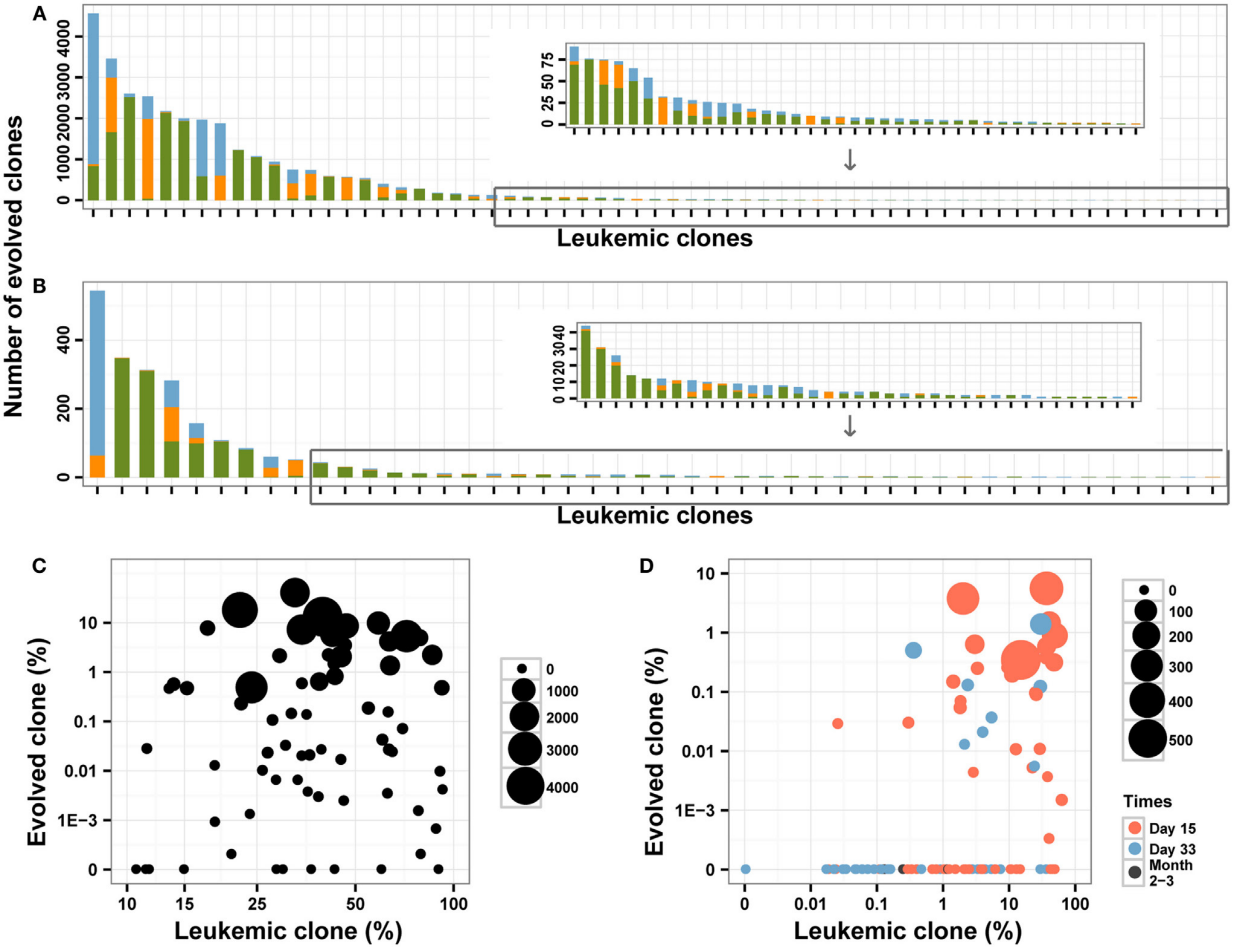
**The evolved clones in pre-treatment and post-treatment bone marrow (BM) samples**. **(A,B)** The number of evolved clones in pre-treatment **(A)** and follow-up **(B)** BM samples. Different colors indicate different relative position (green for upstream, orange for downstream, and blue for uncertain) for the evolved clonotypes compared to the leukemic clones. **(C,D)** The correlation between the leukemic clonal frequencies and the evolved clones in pre-treatment **(C)** and post-treatment **(D)** samples. The size of the point indicates the unique number of evolved clones from each leukemic clone, and colors in **(D)** indicated the time of BM obtained after the beginning of chemotherapy.

To explore the roles of these mechanisms, we assessed and compared the V segments used and the CDR3 length between the leukemic clones and the evolved *IGH* clones. In total, 51.62% of evolved *IGH* clones were mainly replaced by upstream V genes compared to 19.67% by downstream V segments (Figures 4A,B). For the evolved *IGH* clones using upstream V segments, most of the CDR3s were longer than the corresponding leukemic clones; however, the CDR3 lengths of the evolved *IGH* clones using downstream V segments were comparable with the leukemic clones (Figure S3 in Supplementary Material). The above result demonstrated that the evolved *IGH* clones we identified were produced mainly by the first mechanism; in addition, the third mechanism also contributed to the evolved *IGH* clones. Because the pre-leukemic clones were also important in leukemic development and could lead to relapse of disease, we included all those evolved *IGH* clones in subsequent analysis.

In the diagnostic BM samples, we identified evolved *IGH* clones in most of the patients, and only two patients did not have evolved *IGH* clones. We also found that four leukemic clones were produced due to V gene replacement of the other four leukemic clones in three patients (Table S2 in Supplementary Material). The number of identified evolved *IGH* clones varied from 0 to 4558 for each leukemic clone (Figure 4A), with 14 (18.2%) of the leukemic clones did not experience *IGH* evolution. When investigating the ORF of the evolved *IGH* clones, only 11.5% of those clones were functional with correct ORF, which is much lower than the proportion of the leukemic clones. The total frequency of these clones varied from 0 to 38.55% for each leukemic clone (Figure 4C). There was a positive correlation between the total frequency and the number of evolved *IGH* clones for each leukemic clone (Pearson’s *r* = 0.6661; Spearman’s *r* = 0.9851). Whether the number and frequencies of the evolved *IGH* clones were determined by the total number of the leukemic cells is an interesting question. We found that both the number (Pearson’s *r* = −0.0490) and total frequencies (Pearson’s *r* = −0.0985) of evolved *IGH* clones was not associated with the frequency of the cancer clone (Figure 4C). The association of evolved *IGH* clones with clinical features is another important question; however, we did not identify their correlation with disease characteristics in our dataset (Figures S4A,B in Supplementary Material).

We next investigated the dynamic change of the evolutionary *IGH* clones following chemotherapy treatment in BM. Forty-six percentage of the MRD positive samples contained evolved *IGH* clones, and we did not detect evolved *IGH* clones in MRD negative samples (Figure 4D). We also investigated the ORF of these evolved *IGH* clones, and the results showed only 12.7% of those clones were functional with correct ORF, which was similar to the proportion of evolved *IGH* clones in diagnostic samples. The total frequency of these evolved *IGH* clones showed very weak correlation with the frequency of leukemic clones (Pearson’s *r* = 0.2203, Figure 4D). Tracking the fluctuation of frequency ratios of the evolved *IGH* clones to the leukemic clones revealed the imbalanced response of leukemic clones and evolved *IGH* clones to chemotherapy. The ratios of total frequencies of evolved *IGH* clones to the frequencies of leukemic clones decreased during treatment for most of clones; however, for several clones, the day 15 samples showed higher ratio than the diagnostic samples (Figure S5 in Supplementary Material), which may imply positive selection of the evolved *IGH* clones upon the pressure of chemotherapy in the begin of treatment. Interestingly, we found a significant number of evolved *IGH* clones newly emerged in the post-treatment samples (Figure S6 in Supplementary Material), even though these clones eventually disappeared with the negative detection of MRD after chemotherapy.

**Figure 5:**
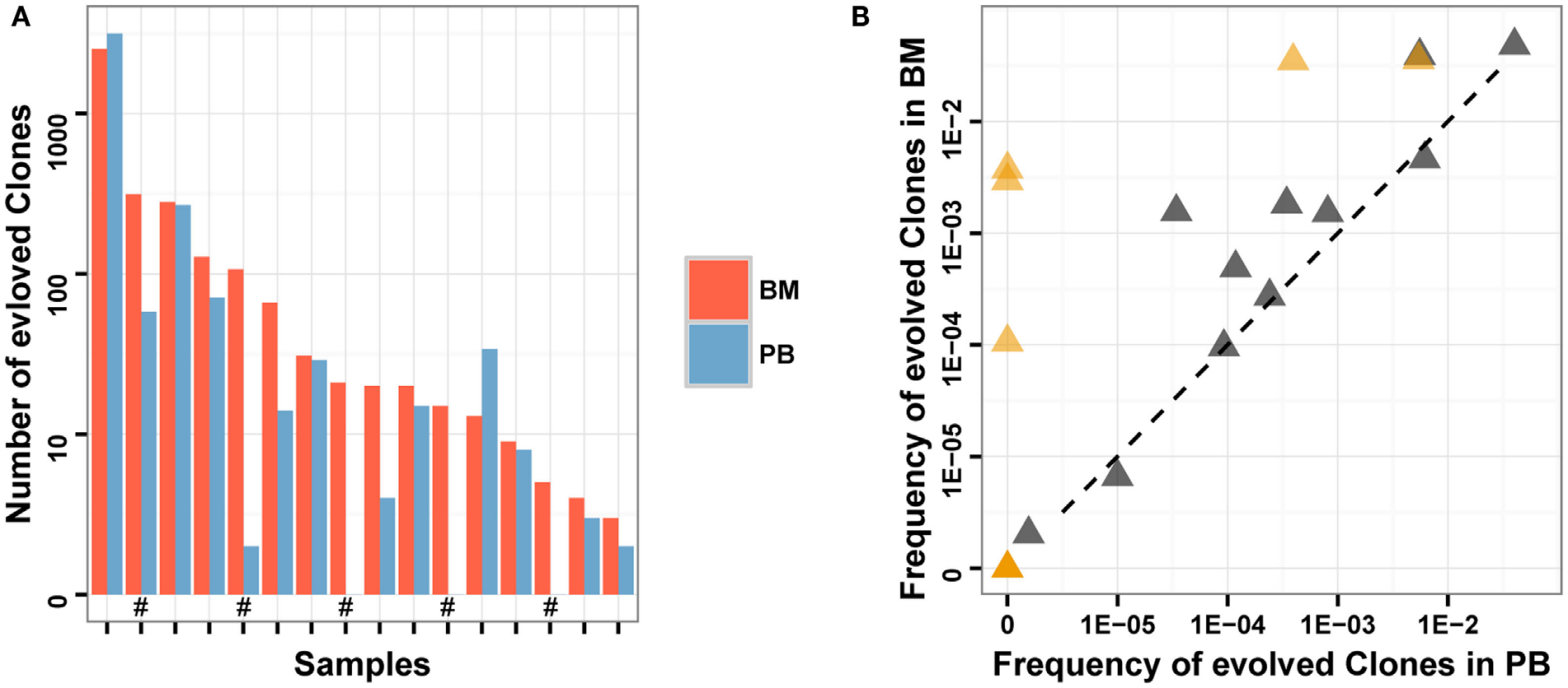
**Comparison of evolved clones in PB and BM**. **(A)** The number of evolved clonotypes identified in PB and BM of the same samples. “#” in the *X*-axis indicates follow-up samples. **(B)** The total frequency of evolved clones in PB and BM of the same samples. Brown triangles indicate follow-up samples.

We compared the number and total frequency of evolved *IGH* clones in PB and BM of the same patients. The result showed that the number and total frequency of evolved *IGH* clones in PB were correlated positively with that in BM (Pearson’s *r* = 0.9941 for number; Pearson’s *r* = 0.7154 for total frequency). However, for most samples, the total frequency of evolved *IGH* clones in PB was lower than that in BM (Figure 5). This result revealed that PB has the potential to serve as a non-invasive specimen to monitor clonal *IGH* evolution during therapy, but it may underestimate the frequency of evolved *IGH* clones.

## Discussion

Leukemia is a clonal disease, and pediatric B-ALL is derived from an early stage of B cell development. During early B cell development, the germline variable (V), diversity (D) and joining (J) gene segments of Ig recombine and, therefore, each B cell obtains a particular combination of V–D–J segments (30). The random deletion at the end of germline segments and the random insertion of nucleosides between the V–D and D–J gene segments during rearrangement creates huge diversity of CDR3 of the *IGH* gene. According to the above production mechanism of the antibody diversity, the CDR3 sequence of the *IGH* can be used as a unique proxy to distinguish different B cells. *IGH* sequence has been used as a tumor-specific biomarker to detect MRD for decades by conventional polymerase chain reaction (PCR) or RQ-PCR (7, 32, 33). Since the development of immune repertoire HTS technology in 2009 (34–36), several studies have tried to detect MRD of pediatric ALL, including T-ALL (36) and B-ALL (17, 18) using this technology. In order to evaluate the wider applicability of this technology, we detected the MRD of B-ALL patients with different treatment days using immune repertoire HTS. We also investigated the evolved *IGH* clones in pre-treatment and post-treatment samples.

We identified clonal *IGH* rearrangements in 92.2% of the unselected cohort of childhood B-ALL patients (Table 1). The four patients without clonal *IGH* rearrangement detected did not show any difference in clinical phenotype and FCM result. It is possible that these leukemic cells were in an earlier developmental stage in those patients, just finished the D–J rearrangement, but not the complete V–D–J rearrangement, which has been observed in previous studies (17, 18, 37). It is also possible those leukemic cells were in the earliest recognizable B-lineage cells, which did not begin the rearrangement. Previous study had also reported that most of the childhood B-ALL (>83%), but not all the patients, had *IGH* gene rearrangement (7, 33). Therefore, even though the high sensitivity of MRD detection using HTS, the limitation in failure to diagnose the pro-B-ALL, which consists of about 10% of B-ALL patients, indicated that FCM was still needed to accurately diagnose B-ALL and detect MRD for those patients.

Importantly, we found that disease risk of B-ALL is related to the number of leukemic clones in diagnostic samples for the first time. Concretely, sample with more leukemic clones indicates higher disease risk, which means slower reaction to chemotherapy reagent (Figure 1). More leukemic clones may imply higher RAG1 and RAG2 activity, which could not only target the Ig gene but also the non-Ig gene (such as tumor suppressor gene IKZF1, CRLF2, BTG1, etc.), and lead to the genomic instability according to Swaminathan et al. (23). They reported that high RAG1 mRNA expression predicted poor ALL patient outcome, which could explain our result.

During the V–D–J rearrangement of *IGH* in B cell development, the random deletion and addition of nucleotides between V–D and D–J segments at the time of joining could result in variability at the junctions and different ORF in CDR3. The possibility that the length of the CDR3 is exact multiple of three nucleotides is one-third expected from random rearrangement. Therefore, in theory, only one in three *IGH* rearranged B cells could make an in-frame and functional rearrangement before selection, which has been demonstrated previously (38). In our study, about one-third of the disease clones had in-framed CDR3 ORF, and two-thirds of the disease clones had frame-shift mutation in the CDR3 (Table S2 in Supplementary Material). Therefore, our data are consistent with random recombination and suggested that leukemic cells were in the very early development stage without experiencing positive selection.

In the normal B cell development, precursor-BCR (pre-BCR) checkpoint controls critical B-cell developmental processes and is essential to make sure that the B cells develop into mature B cell (39–41). The pre-BCR has to be expressed on the cell surface to induce the B cell to pass this checkpoint. In order to pass the checkpoint, the B cell should complete in-frame *IGH* VDJ recombination to express a functional Pre-BCR. If a precursor B cell possesses an out-of-frame *IGH* rearrangement, there is no pre-BCR expressed on the surface of the B cell, therefore, the cell does not receive necessary survival signals, and it will undergo a programed cell death. In our study, we found that two-thirds of the leukemic cells do not have functional *IGH* V–D–J rearrangement (Table S2 in Supplementary Material), but they survived the selection and proliferated to clonal cells. A similar result was also observed in another study in *BCR-ABL1* positive B-ALL cells (42), which reported only 3 out of 12 *BCR-ABL1* positive B-ALL cases harbored potentially functional *IGH* gene rearrangements. Several studies have reviewed that leukemic B cells could manage to evade the pre-BCR checkpoint and avoid clonal extinction by mimicing or bypassing the Pre-BCR signaling pathway (43–45).

As to MRD detection, our data demonstrated the robustness of HTS similar to previous studies (16–18). Compared to FCM, HTS did not show false-negative results, and the HTS method can detect MRD at levels lower than 0.01%, which cannot be detected by FCM (Figure 2). In general, our study confirmed the wide applicability of HTS in MRD detection and higher sensitivity than FCM. There were eight post-treatment samples, whose MRD were higher than 0.1% by HTS, but were reported negative in the FCM detection. Wu et al. discovered a similar phenomenon in their study, and verified and explained that this is possible due to immunophenotypic normalization induced by therapy (17). Because most the patients in our cohort were followed up for less than 2 years, and no patient relapsed, we cannot assess the clinical significance of the higher MRD detection sensitivity using HTS in this panel. Wu et al. discussed the prognostic significance by reviewing previous studies, and concluded that very low-level MRD detected by HTS is likely to be meaningful and, therefore, they suggests that these patients should be closely monitored (17).

To date, BM is the most used specimen for MRD detection in leukemia; however, it has been proposed that PB might represent a more convenient specimen for monitoring MRD. In this study, we found that for samples with relatively high leukemic clone frequency in BM (approximately above 1%), PB samples could detect MRD but with relatively lower MRD levels than BM samples (Figure 3), and this result agreed with the Coustan-Smith et al.’s study in B-ALL (20). Overall, there is a false-negative rate and the MRD level could be underestimated in PB, which limited the application of PB in clinic for MRD detection today. Larger cohort with more comprehensive design is needed to determine how PB sample should be used directly in clinic. For example, the MRD level in PB 7 day post-treatment may correlate well with the MRD level in BM 15 day post-treatment; therefore, it is possible we could use the MRD level in PB 7 day post-treatment to predict the prognosis of leukemia. Notably, in our study, one negative leukemic clone in BM was found to be positive in PB, which implied PB might provide compensated prognostic value to BM.

Clonal evolution is an important question in leukemia development and treatment. In this study, we identified evolved *IGH* clones induced by V replacement, and also the correlation of those clones with the frequencies of leukemic clones in diagnostic BM samples (Figure 4C). More importantly, we found that four leukemic clones were produced due to V gene replacement from other leukemic clones (Table S2 in Supplementary Material), which suggested that evolved *IGH* clones could expand greatly and lead to disease relapse if not eliminated during treatment. We also investigated the reaction of those evolved *IGH* clones during chemotherapy. Most of the day 15 and part of the day 33 post-treatment samples contained evolved *IGH* clones (Figure 4D) and abundant new evolved *IGH* clones not present in the diagnostic samples emerged upon the pressure of therapy (Figure S6 in Supplementary Material), which demonstrated that chemotherapy can induce continuous evolution of the leukemic cells and increase the heterogeneity of cancer. When investigating the response of evolved *IGH* clones to chemotherapy, both the clones identified in pre-treatment samples and those newly generated during treatment decreased during the course of therapy and disappeared when the MRD was negative (Figure 4D). Therefore, those evolved *IGH* clones were also therapy sensitive in primary cancer, which indicate good prognosis of those patients. However, we cannot make sure if a clone harboring an evolved *IGH* gene actually is to be equaled to the generation of a new clone with new genomic variants. In order to validate this correlation, single-cell sequencing used by Gawad et al. (46) is required to investigate the genome-wide somatic variants and the *IGH* DNA sequence of each single cell.

In this paper, we confirmed the wide applicability and higher sensitivity of HTS in MRD detection of B-ALL patients. Considering the invasiveness of BM extraction, we assessed the value of using PB as MRD detection specimen, and suggested that although MRD in PB could not represent the exact MRD level of the patients, PB could be used to evaluate the response of cancer cells to chemotherapy in the beginning of the treatment. During the disease development and disease treatment, cancer cells could evolve and generate high genetic heterogeneity of genome, including *IGH* gene. We identified many evolved *IGH* clones in the diagnostic samples. However, nearly all those evolved *IGH* clones decreased with on-going treatment and disappeared when the MRD became negative in these samples. It is possible some of those evolved *IGH* clones could lead to relapse of the disease.

## Data Accession

The raw data has been deposited to Sequence Read Archive (SRA) under accession SRA456729.

## Author Contributions

CL and XL designed the study and supervised research. JW and CW performed research, analyzed data, and wrote the paper. SJ collected the specimens and clinical information. WZ, YD, XL, and XX analyzed the data. XZ and XH performed the experiments. SL, HM, XY, XW, and FW contributed samples. JP checked the FCM MRD detection result. All authors contributed to the preparation of the manuscript and approved the submission in its current form.

## Conflict of Interest Statement

JP has employment with KingMed Diagnostics. All the other authors declare no competing financial interests.

## Abbreviations

ALL, acute lymphoblastic leukemia; B-ALL, B cell lineage ALL; BM, bone marrow; CDR3, complementarity determining region 3; FCM, flow cytometry; FR3, framework region 3; HTS, high throughput sequencing; IGH, immunoglobulin heavy chain; MRD, minimal residual disease; ORF, open reading frame; PB, peripheral blood; RQ-PCR, real-time quantitative polymerase chain reaction; TCR, T cell receptor.
