# Supplementary material for: Minimal Residual Disease Detection and Evolved IGH Clones Analysis in Acute B Lymphoblastic Leukemia Using IGH Deep Sequencing
Source: Front Immunol. 2016 Oct 4;7:403. doi: 10.3389/fimmu.2016.00403 (PMC5048610; doi:10.3389/fimmu.2016.00403)
Supplement: Supplementary file 1 [file Data_Sheet_1.DOCX]

Supplementary Material

Minimal residual disease detection and evolved *IGH* clones analysis in acute B lymphoblastic leukemia using *IGH* deep sequencing

**Jinghua Wu, Shan Jia, Changxi Wang, Wei Zhang, Shixi Liu, Xiaojing Zeng, Huirong Mai, Xiuli Yuan, Yuanping Du, Xiaodong Wang, Xueyu Hong, Xuemei Li, Feiqiu Wen, Xun Xu, Jianhua Pan, Changgang Li^*^, Xiao Liu^*^**

***Correspondence**:

Xiao Liu,

liuxiao@genomics.cn

Changgang Li,

licg6336@sina.com

1. **Supplementary Figures and Tables**

## Supplementary Figures

**Figure S1.** Comparison the IGH RNA transcription level between functional and non-functional leukemic clones, and no significant differences were detected (P = 0.4462). The RNA transcription level was reflected by the rate of the clonal frequency in RNA sample to that in DNA sample.

**Figure S2.** The dynamic change of leukemic clone frequencies along with the treatment progressed.

**Figure S3.** Comparison the CDR3 length of evolved clones and leukemic clones for evolved clones using upstream V segment (A) and downstream V segment (B). Different colors indicate the number of evolved clones with the corresponding CDR3 length.

**Figure S4.** Association of total frequency (A) and number (B) of V-replaced clones with disease risk in diagnostic sample. HR, high risk; IR, intermediate risk; SR, standard risk. P value was calculated by Mann-Whitney U test.

**Figure S5.** The changing ratio of the total V-replaced clones’ frequency to leukemic clone frequency with treatment. Just the MRD positive samples were plotted.

**Figure S6.** The constitution of evolved leukemic clones induced by V-replacement in post-treatment born marrow samples. Blue indicated primary clones existing in diagnostic samples; Red indicated new clones emerging in post-treatment samples.

## Supplementary tables

**Table S1.** Specimen collection information.

| Patients ID |  |  |  |  | Specimen collection time | | | |
| --- | --- | --- | --- | --- | --- | --- | --- | --- |
|  | Age | Gender | Risk group | Cytogenetics | Day 0 | Day 15 | Day 33 | Day 60-100 |
| P001 | 1.7 | F | SR | Normal | ● |  |  |  |
| P002 | 7 | M | HR | Normal | ● |  |  | ● |
| P003 | 4.3 | M | SR | TEL-AML1 | ● |  | ● | ● |
| P004 | 1.5 | F | IR | Normal | ●▲ |  | ● | ● |
| P005 | 2.2 | F | HR | MLL-AF4 | ●▲ |  | ● |  |
| P006 | 4.2 | F | SR | Normal | ● | ● | ● |  |
| P007 | 3.7 | M | SR | TEL-AML1 | ●▲ |  | ● | ● |
| P008 | 2.7 | F | IR | Not done | ●▲ | ● | ● |  |
| P009 | 1.9 | F | SR | Normal | ● |  | ● |  |
| P010 | 2.2 | M | IR | Not done | ● |  | ● |  |
| P011 | 2.5 | M | IR | TEL-AML1 | ●▲ | ● |  |  |
| P012 | 8.7 | M | HR | Normal | ● | ● | ● |  |
| P013 | 2 | M | SR | Normal | ● |  | ● | ● |
| P014 | 3.4 | M | SR | Normal | ▲ | ● | ● |  |
| P015 | 1.6 | F | HR | MLL-AF4 | ● | ● | ● |  |
| P016 | 5.3 | M | SR | Normal | ● |  | ● |  |
| P017 | 10.1 | M | HR | TEL-AML1 | ● | ● | ● |  |
| P018 | 3.5 | M | HR | Normal | ▲ | ● | ● |  |
| P019 | 1.9 | F | IR | Normal | ● | ● | ● |  |
| P020 | 1.6 | M | IR | Normal | ● |  | ● |  |
| P021 | 1.2 | F | HR | MLL-AF4 | ▲ | ● | ● |  |
| P022 | 2.4 | M | HR | TEL-AML1 | ● | ● | ● |  |
| P023 | 5 | M | HR | Normal | ● | ● |  |  |
| P024 | 1.7 | M | HR | Normal | ● |  | ● |  |
| P025 | 7.6 | M | IR | Normal | ● |  | ● |  |
| P026 | 3.8 | M | SR | 45, XY, der (7:9) (q10;q10) [4] / 46,XY [4] | ● | ● | ● |  |
| P027 | 2.3 | F | SR | Normal | ● |  |  | ● |
| P028 | 6.8 | M | IR | Normal | ●▲ | ● |  |  |
| P029 | 1.1 | M | HR | MLL-AF4 | ●▲ | ●▲ |  | ● |
| P030 | 1.2 | M | IR | Normal | ●▲ | ●▲ | ●▲ | ● |
| P031 | 1.6 | F | SR | Normal | ●▲ |  | ●▲ | ● |
| P032 | 9.8 | F | IR | TEL-AML1 | ● | ● |  |  |
| P033 | 1.1 | M | IR | Normal | ●▲ | ●▲ | ● |  |
| P034 | 2 | F | SR | Normal | ● | ● | ● |  |
| P035 | 2.3 | F | SR | TEL-AML1 | ● | ●▲ | ●▲ |  |
| P036 | 2.4 | M | IR | TEL-AML1 | ●▲ | ● | ●▲ |  |
| P037 | 3.3 | M | SR | TEL-AML1 | ●▲ | ●▲ | ●▲ | ● |
| P038 | 6.9 | F | IR | Normal | ●▲ | ●▲ | ●▲ |  |
| P039 | 6.1 | M | HR | TEL-AML1 | ● | ● | ● | ● |
| P040 | 6.9 | M | IR | Not done | ● | ● | ● |  |
| P041 | 11.3 | M | IR | Not done | ● | ● | ● |  |
| P042 | 2.5 | M | HR | Not done | ● | ● |  | ● |
| P043 | 4.5 | M | IR | 51, XY, +X, +4, +6, ins (14;?) (q13;?), +17, +21 [4] /52, idem, +18 [3]/46, XY [3] | ●▲ | ●▲ | ● |  |
| P044 | 2.7 | F | SR | Normal | ● | ● | ● |  |
| P045 | 3.3 | M | SR | TEL-AML1 | ● | ● | ● |  |
| P046 | 5.6 | M | HR | TEL-AML1 | ● | ●▲ | ● |  |
| P047 | 5.5 | F | SR | Normal | ● | ● | ● |  |
| P048 | 3.6 | F | SR | Normal | ●▲ | ● | ●▲ |  |
| P049 | 8.2 | M | IR | E2A-PBX1 | ● | ● | ● |  |
| P050 | 2.4 | M | SR | Not done | ● | ● |  |  |
| P051 | 1.7 | M | IR | Normal | ● | ● |  |  |

The ● refers to born morrow specimen, and ▲ refers to peripheral blood sample. SR = Standard Risk; IR = Intermediate Risk; HR = high risk

**Table S2.** Summary of the leukemic clones and HTS MRD detection result.

|  |  | Diagnostic samples | | | Following up samples MRD | | |
| --- | --- | --- | --- | --- | --- | --- | --- |
| Patients ID | Leukemic clone ID | CDR3 sequence of leukemic clones | ORF | Frequency of leukemic clones (%) | day15 | day33 | day 64-92 |
| P001 | P001A | TGTGCGAGACGAAGAGGGTATTACTATGATAGTAGTGGTTATCTCTAACTCCTACTACTACTACGGTATGGACGTCTGG | frame shift | 15.4207 | NA | NA | NA |
|  | P001B | TGTGCGGGGGATTATTCCTATGATAGTAGCGGTCTACTACTACTACGGTATGGACGTCTGG | frame shift | 35.7939 |  |  |  |
|  | P001C | TGTGCAAAAGATAGGGGAACGAAGAGGGTATTACTATGATAGTAGTGGTTATCTCTAACTCCTACTACTACTACGGTATGGACGTCTGG | frame shift | 14.8034 |  |  |  |
|  | P001D | TGTGCGAAAGATGGAAGATCGTAAAGGGTTACTATGATAGTAGCGGTCTACTACTACTACGGTATGGACGTCTGG | stop codon | 14.8243 |  |  |  |
| P002 | P002 | TGTGCGAGAGAGACCCGGGCCCTACGTATTACGATATTTTGACTGGTTATTATATTTCCCAGACTACTGG | frame shift | 69.8609 | NA | NA | 0.0000 |
| P003 | P003 | TGTGCGGTGACTGCTATTTGACTACTGG | frame shift | 84.5640 | NA | 0.0093 | 0.0015 |
| P004 | P004 | CCTCGGAGGTAGTACCAGCTGCTATGCCCGGTGGTTCGACCCCTGG | frame shift | 78.6172 | NA | 0.0000 | 0.0000 |
| P005 | P005A | TGTGCAAGAGATTGTAGTAGTACCAGCTGCCCCCTGAACTGGGGACTACTACGGTATGGACGTCTGG | frame shift | 45.5225 | NA | 0.1299 | NA |
|  | P005B | TGTGCGAGACATGGGGGATATTGTAGTAGTACCAGCTGCCCGTCTTCGACCCCTGG | frame shift | 40.9627 |  | 0.1004 |  |
| P006 | P006 | TGTGCGAGAGGGCATTGTAGTAGTACCAGCTGCTATGGTACTTCGATCTCTGG | frame shift | 90.8681 | 0.0112 | 0.0532 | NA |
| P007 | P007 | TCCCTGGGCCTATATTGTACTAATGGTGTCGCCTGG | correct | 87.5892 | NA | 0.0000 | 0.0000 |
| P008 | P008A | TGTGCGAGAGTCCCTGTATAGCAGCTCGTCCGGTGGTGGCTGG | frame shift | 62.7993 | 2.1557 | 0.0000 | NA |
|  | P008B | TGTGCGAGAGTTCGGATGGTTCAGGGAGTTATTTGCACTACTGG | frame shift | 18.4354 | 1.4209 | 0.0000 |  |
|  | P008C | CCTAGCAGCTCGTCCGGTGGTGGCTGG | correct | 11.5988 | 1.2561 | 0.0000 |  |
| P009 | P009A | TGTGCGAGAGATCCTGTTCGGGGAGTTATTATGCTTTTGATATCTGG | frame shift | 38.2418 | NA | 0.0062 | NA |
|  | P009B | GAGTCTATGGTTCGGGGAGTTATTATGCTTTTGATATCTGG | frame shift | 42.8807 |  | 0.0082 |  |
| P010 | P010 | TGTGCTCCAGTGTAGCAGCAGCTGGTACAAACTACTGG | frame shift | 25.1197 | NA | NA | NA |
| P011 | P011A | TGTGCGAGACCGGGGCGAGTATAGCAGCCCGTCAGGACTACTACTACGGTATGGACGTCTGG | frame shift | 44.3996 | 0.3869 | NA | NA |
|  | P011B | TGTGCAAGTTTAGTTCTTTGGAGTGGTTATTATACTACTACTACTACATGGACGTCTGG | frame shift | 38.2008 | 1.4884 |  |  |
| P012 | P012A | TGTGCGAGAGTGGGAGGGGGTTACTACTACTACTACGGTATGGACGTCTGG | correct | 27.6444 | 10.8020 | 0.0000 | NA |
|  | P012B | TGTGCGAGAAAGAGTAAAGGTATGGACGTCTGG | correct | 10.5902 | 4.6961 | 0.0000 |  |
| P013 | P013 | TGTGCGAGAGATAGGGGCTAGGGTCCCAGTTTGACTACTGG | frame shift | 91.7288 | NA | 0.0769 | 0.0000 |
| P014 | P014 | TGTGCAAAAGATCCTCCTTCCAGTGGTGGTAGCTGCTACTCGTGCTTTTGATATCTGG | frame shift | 15.0847 | 0.0454 | 0.0000 | NA |
| P015 | P015 | TGTGCAAGAGACTGGAACTACTTTGACTACTGG | correct | 36.3769 | 6.4527 | 2.6492 | NA |
| P016 | P016 | TGTGCACTCTTTGGGAGAGGCCTCTTAACTGGGGATCTCGGGGGCTTCGATCTCTGG | correct | 45.8412 | NA | 0.0000 | NA |
| P017 | P017A | TGTGCGAGAGCCATGGGGCCCTACTAGTACATGGACGTCTGG | stop codon | 26.7020 | 0.3449 | 0.0000 | NA |
|  | P017B | TGTGCGAGAGCCCCCCCGTTCAATAGCAGCAGCTGAAGGGGACTGGTTCGACCCCTGG | frame shift | 42.7011 | 15.2510 | 0.1077 |  |
| P018 | P018A | TGTGCAAAAGATAGCCCCCCATATTGTTAGGGTACTACTACTACGGTATGGACGTCTGG | frame shift | 33.4311 | 6.5223 | 2.1874 | NA |
|  | P018B | TGTGCAAGAGAGGAATGTAGTAGTACCAGCTGCTTCCCGGCGGTATGGACGTCTGG | frame shift | 34.5318 | 6.0448 | 2.2320 |  |
| P019 | P019A | TGTGCGAGAGATCAGAGGGTTCGGGGAGTTCCCATATCGGGGACTGGTTCGACCCCTGG | frame shift | 64.3404 | 0.2337 | 0.0000 | NA |
|  | P019B | TGTGCGGGTAACTGCCTTAAGCGGCCAGCCCCCGACGGGGCTTTTGATATCTGG | correct | 25.7726 | 0.1192 | 0.0000 |  |
| P020 | FI | FI | FI | FI | NA | FI | NA |
| P021 | P021A | TGTGCAAGATTTGTAGCAGCTCGACAACTGGTTCGACCCCTGG | frame shift | 35.1776 | 17.5761 | 3.1972 | NA |
|  | P021B | TGTGCAAGAGATGGCAAGTGTGGTACTTTGACTACTGG | frame shift | 33.9915 | 20.0409 | 3.9486 |  |
| P022 | P022A | TGTGCGAGAGATAAATTGTAGTGGTGGTAGCTGCTACCCATCTCTAACTAAGCTAACTGGGGCCCTCGGGGGGACTACTGG | stop codon | 38.4091 | 0.2759 | 0.0817 | NA |
|  | P022B | TGTGCGAGAGGCCAAGGGAACTGGGGATTTGGCTGGTTCGACCCCTGG | correct | 17.4350 | 0.0037 | 0.0000 |  |
| P023 | P023A | TGTCCCCAAAAGATAGTATTTTGACTGGTTAACTACTGG | stop codon | 62.2146 | 9.5977 | NA | NA |
|  | P023B | TGTGCGAGAACTACGGTGGTAACTGGGGGCTACTTTGACTACTGG | correct | 28.9774 | 0.5050 |  |  |
| P024 | P024 | TGTGCGAGAGATACGACCTAACTGGCGCCGTAATTTGACTACTGG | stop codon | 59.9760 | NA | 0.3853 | NA |
| P025 | FI | FI | FI | FI | NA | NA | NA |
| P026 | P026 | TGTGCGAGAGGGGTGGGCCTGGAACTCTGAACCACTGGGGGTGTCGTACAAGGGTACTACTACTACATGGACGTCTGG | stop codon | 39.0282 | 0.0243 | 0.0008 | NA |
| P027 | P027A | TGTGCGAGACTGGAAAGGCTAGGTACTATGGTTCGGGGAGTTGGGGGTGATGCTTTTGATATCTGG | correct | 42.6506 | NA | NA | 0.0106 |
|  | P027B | TGTGCAAAAGATAAGGGGGGATTGTAGTAGTACCAGCTTTAGACTGGTTCGACCCCTGG | frame shift | 21.4878 |  |  | 0.0051 |
| P028 | P028 | TGTGCAAGGTCCCCACGGGATAGTAGTGGTTTCCCTTTGACTACTGG | frame shift | 23.4257 | 2.7760 | NA | NA |
| P029 | P029A | TGTGCGAGATCCATCCCCTTTGACTACTGG | correct | 59.6752 | 6.8208 | NA | 0.0000 |
|  | P029B | TGTGCAAGAGATAAGGGTTCGGGGACCTTCCAGCACTGG | correct | 20.7051 | 0.5375 |  | 0.0000 |
| P030 | P030 | TGTGCGAGAGTGGGTATAGCAGCAGCTGGTCCTAACTGGTACTTCGATCTCTGG | correct | 77.6665 | 4.3320 | 0.0037 | 0.0000 |
| P031 | P031A | TGTGCTCCCCCTATTGTAGTGGTGGTAGCTGCTGACTCCGGAGTAGTACTACTTTGACTACTGG | frame shift | 31.5578 | NA | 0.0028 | 0.0000 |
|  | P031B | TGTGCGAGAGATCCCAGTCACTACTACTACTACATGGACGTCTGG | correct | 18.4054 |  | 0.0016 | 0.0000 |
| P032 | FI | FI | FI | FI | FI | NA | NA |
| P033 | P033 | TGTGCGAGATCCCATTCCATATCCGTCACTATGTACTACTACATGGACGTCTGG | correct | 62.4427 | 0.0705 | 0.0000 | NA |
| P034 | P034A | TGTGCAAGAGGGGTACTATGGTTCGGGGACCCTCTACTACTACTACGGTATGGACGTCTGG | frame shift | 33.0476 | 0.0015 | 0.0000 | NA |
|  | P034B | TGTGCGAGAGACCGGGGGTGGTTCGACCCCTGG | correct | 29.7941 | 0.0019 | 0.0000 |  |
| P035 | P035A | TGTGCGACCCGGTATAGCAGCAGCTGGACTTTGACTACTGG | frame shift | 37.6729 | 0.5424 | 0.0018 | NA |
|  | P035B | TGTGCAAAAGATGGGGGGAGAGACCCTTATAGCAGCAGCTGGACTTTGACTACTGG | frame shift | 15.8163 | 0.0054 | 0.0000 |  |
| P036 | P036A | TGTGCGAGAGACGACCTACGCTCGGGCAGGGGGTTCGGGGAGTTATACTACTACTACGGTATGGACGTCTGG | correct | 62.8079 | 1.6286 | 0.0052 | NA |
|  | P036B | TGTGCGAGACTTAACGGGATGCCGGGCGGGTACGTCCATAACTGGGGATCGCCATGACTACTGG | frame shift | 22.1055 | 0.0438 | 0.0000 |  |
| P037 | P037 | TGTGCGAGAAAGGAGCCACTACTACTACGGTATGGACGTCTGG | frame shift | 89.2140 | 5.1336 | 0.1193 | 0.0192 |
| P038 | P038A | TGTGCGAAAGATAGATGGTTCGGGGAGTCTCCCTCAACTGGGGAGGACTACGGTATGGACGTCTGG | correct | 30.3215 | 0.1654 | 0.0000 | NA |
|  | P038B | TGAGGTAGCAGGGGATTGTGGTGGTGACTGCTATTCCGGCTACAACTGGTTCGACCCCTGG | frame shift | 44.7524 | 0.2789 | 0.0010 |  |
| P039 | P039A | TGTGCGAGAGATAGTGAGCTAACTACTACTACGGTATGGACGTCTGG | frame shift | 45.9529 | 16.4671 | 0.0018 | 0.0000 |
|  | P039B | TGTGCGATGCGGAGGGCTAACTACTACTACGGTATGGACGTCTGG | correct | 13.3430 | 4.6268 | 0.0000 | 0.0000 |
| P040 | P040A | TGTGCGAGATGTACCCCCCCACTACCTTAGAGAGACTACTGG | stop codon | 41.6787 | 10.7586 | 0.2326 | NA |
|  | P040B | TGTGCGAGAGGCTACTACTACGGTATGGACGTCTGG | correct | 14.8360 | 0.5793 | 0.0050 |  |
|  | P040C | TGTGCGGCAGATTGGAGGTGGTTCGACCCCTGG | correct | 11.3345 | 0.9250 | 0.0069 |  |
|  | P040D | TGTGCGAGAGATCTCGGCTGGTCCAACTGGTTCGACCCCTGG | correct | 11.4092 | 0.4248 | 0.0000 |  |
| P041 | P041A | TGTGCAACTATTACTACTACTACTACTACATGGACGTCTGG | frame shift | 28.4619 | 0.0910 | 0.0000 | NA |
|  | P041B | TGTGCGAGAGGTAACGTGCAGCAGCTGATTAGCTTTGACTACTGG | correct | 23.5760 | 1.0228 | 0.0000 |  |
|  | P041C | GGTCCCGTTGACTACGGTGGTACTAGAATTTGACTACTGG | frame shift | 28.3630 | 10.3234 | 0.0018 |  |
| P042 | P042 | TGTGCGAGAGATCAAACTGGGCTGCATTACTTTGACTACTGG | correct | 33.9937 | 0.7955 | NA | 0.0000 |
| P043 | P043 | TGTGCAAAAGAAGGGGACTGGGGACGACCTACTACTACTACTACGGTATGGACGTCTGG | frame shift | 54.2512 | 3.3941 | 0.7177 | NA |
| P044 | P044 | TGTGCAAAAGATACTATTACGATTTTTGGAGTGGTTACGTGCCGCTACTACTACTACTACTACATGGACGTCTGG | correct | 35.9186 | 0.0773 | 0.0000 | NA |
| P045 | P045 | TGTGCGAGAGACCCGAGAGCAAAGACCTTTCCGGTATAGCAGCTACTACTTTGACTACTGG | frame shift | 62.4613 | 0.6442 | 0.0179 | NA |
| P046 | P046 | TGTGCGAGAGGCGTCGGGGTATGGTGGCTACAATTACTACTACTACTACGGTATGGACGTCTGG | frame shift | 31.8855 | 0.4787 | 0.0263 | NA |
| P047 | FI | FI | FI | FI | FI | FI | NA |
| P048 | P048 | TGTGCGAGAGATCCCTCCTATAGCAGCAGCTGGTGTGTGAACTACTACGGTATGGACGTCTGG | correct | 89.9973 | 0.2050 | 0.0000 | NA |
| P049 | P049A | TGTGCACGGACGAGGGACTCCTCCCCCGTGGTGACTACGTAGGACTACTGG | stop codon | 69.1297 | 3.8294 | 0.0008 | NA |
|  | P049B | TGTGCGAGACAGGGTATAGCAAGGACGGGGGTTGACTACTGG | correct | 13.7537 | 0.1342 | 0.0000 |  |
| P050 | P050 | TGTGCGAAGTGAGAGTGAGGTGGCCCACTTTGACTACTGG | frame shift | 57.7161 | 2.0564 | NA | NA |
| P051 | P051A | TGTGCAAGAGGGAATTGTAGTAGTACCAGCTGCTCCGGGGTGGACGTCTGG | correct | 45.7169 | 0.6991 | NA | NA |
|  | P051B | TCGTGCGTCGATACTGGCAGCAGCTCGGTTTGACTACTGG | frame shift | 35.4801 | 0.2612 |  |  |

NA = No assay; FI = fail to identify leukemic clones; Underline of "frequency of leukemic clones (%)" means using peripheral blood specimen; The same color in "CDR3 sequence of leukemic clones" means one clone produced due to V-replacement of the other clone.

**Table S3.** MRD detection in two experiments for the same samples.

| Samples | Clones | MRD in repeat 1 (%) | MRD in repeat 2 (%) |
| --- | --- | --- | --- |
| P051-15 | leukemic clone 1 | 0.6991 | 0.6865 |
|  | leukemic clone 2 | 0.2612 | 0.2794 |
| P049-15 | leukemic clone 1 | 3.8294 | 3.6630 |
|  | leukemic clone 2 | 0.1342 | 0.0989 |
| P049-33 | leukemic clone 1 | 0.0008 | 0.0000 |
|  | leukemic clone 2 | 0.0000 | 0.0000 |

**Table S4.** Primers used in multiplex PCR for amplifying the CDR3 of rearranged *IGH* gene.

| Primer set | Primer name | Sequence (5’ to 3’) |
| --- | --- | --- |
| Forward Primer | IGHV1-A | AGAGTCACCATGACCACAGAC |
|  | IGHV1-B | AGAGTCACCAKKACCAGGGAC |
|  | IGHV1-C | AGAGTCACCATGACCGAGGAC |
|  | IGHV1-D | AGAGTCACCATTACYAGGGAC |
|  | IGHV1-E | AGAGTCACGATWACCRCGGAC |
|  | IGHV1-F | AGAGTCACCATGACCAGGAAC |
|  | IGHV2 | ACCAGGCTCACCATYWCCAAGG |
|  | IGHV3 | GGCCGATTCACCATCTCMAG |
|  | IGHV4 | CGAGTCACCATRTCMGTAGAC |
|  | IGHV5 | CAGCCGACAAGTCCATCAGC |
|  | IGHV6 | AGTCGAATAACCATCAACCCAG |
|  | IGHV7 | GACGGTTTGTCTTCTCCTTG |
| Reverse Primer | IGHJ1/4/5 | CTGAGGAGACAGTGACCAGGGT |
|  | IGHJ2 | CTGAGGAGACGGTGACCAGGGT |
|  | IGHJ3 | CTGAAGAGACGGTGACCATTGT |
|  | IGHJ6 | CTGAGGAGACGGTGACCGTGGT |
